# Patient Agency in Imaging: Radiologists’ Insights

**DOI:** 10.1002/puh2.70105

**Published:** 2025-08-18

**Authors:** Lizzie De Silva, Melissa Baysari, Melanie Keep, Peter Kench, Jillian Clarke

**Affiliations:** ^1^ Discipline of Medical Imaging Science Sydney School of Health Sciences Faculty of Medicine and Health The University of Sydney, Susan Wakil Health Building, Camperdown Campus Camperdown New South Wales Australia; ^2^ Faculty of Medicine and Health Susan Wakil School of Nursing and Midwifery Sydney Nursing School The University of Sydney Sydney New South Wales Australia; ^3^ Sydney School of Health Sciences Faculty of Medicine and Health, Susan Wakil Health Building The University of Sydney Camperdown New South Wales Australia

**Keywords:** consumerism, doctor–patient relationship, general practitioner, health‐seeking, internet, online health, patient‐requested studies, radiology

## Abstract

**Objectives:**

There is a notable rise in patient‐initiated imaging requests, with one possible reason being the increased availability of online health information. This study specifically examines the impact of patient‐initiated radiological exams on radiologists. Exploring their perspectives on how GPs acquiescing to patients’ medical imaging requests impacts healthcare resources and skew patients’ expectations of the capabilities of radiological studies. The findings of this study are crucial for understanding the evolving dynamics of radiology practice and its challenges.

**Method:**

A comprehensive cross‐sectional opinion survey was administered to radiologists in private multi‐specialty medical centers across Australia between November 2023 and February 2024. The survey included structured and open‐ended questions distributed via an intranet platform within the same company and reached a wide range of radiologists. A total of 37 survey responses were received from the 100 surveys sent to radiologists, resulting in a response rate of 37%. Of these, 10 provided incomplete responses, leaving 27 for analysis.

**Results:**

Nearly half of the participants (48%) had over 20 years of experience. The majority of radiologists self‐reported X‐rays (27%), ultrasounds (25%), and computed tomography (CT) scans (24%) as the most commonly requested non‐indicated imaging studies. Radiologists attributed these requests to patients’ exposure to online medical information and their desire for reassurance. Approximately 24% of radiological studies were deemed unnecessary due to a lack of correlation between clinical history and the imaging or procedure requested to address the clinical issue, with 30% of radiologists reporting feeling pressured by GPs and patients to expedite report completion. Proposed strategies included patient education, communication, and the establishment of clear guidelines.

**Conclusions:**

The study highlights the potential for a growing burden of patient‐initiated imaging on radiologists. This burden is further compounded by patients’ unrealistic expectations and lack of understanding of diagnostic imaging's limitations, as considered by those surveyed. Radiologists have stressed the crucial role of the broader healthcare context in collaborating with such requests. Patient education was emphasisX‐ed to reduce unnecessary imaging and manage patient expectations.

**Practice Implications:**

Educating patients about the limitations and appropriate use of diagnostic imaging can help reduce unwarranted requests and manage patient expectations. The study emphasises the need for clear, evidence‐based guidelines to assist GPs in addressing patient demands for unnecessary imaging studies. Another contribution from the study is the necessity for improved communication strategies among radiologists, GPs, and patients to ensure a collaborative approach to imaging requests, thereby reducing pressure on the radiologists’ workload. Finally, this study highlights how avoiding unnecessary imaging can alleviate resource strain, optimise workflows, and enhance the quality of patient care.

## Graphical abstract

Patients’ exposure to online health information often leads them to seek diagnostic imaging for reassurance, even when it is not clinically justified. GPs, acting as gatekeepers, feel pressured by such patient demands, as fears of litigation and the need to meet patient expectations complicate their gatekeeping role. The study found that 24% of scans were self‐reported as unnecessary by surveyed radiologists, with 30% indicating they were under increased pressure to expedite reports rapidly to appease anxious patients seeking results from their studies. The study concluded that collaboration among health providers is essential for managing patient expectations, reducing unnecessary imaging, and protecting health resources. These measures could be implemented through patient education, adherence to evidence‐based guidelines, and improved multidisciplinary collaboration communication.

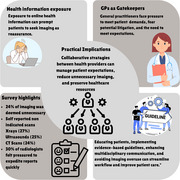

## 1 Introduction

In modern medicine, diagnostic testing has increasingly become popular [1]. These diagnostic tests include laboratory tests, medical imaging, and invasive procedures such as image‐guided biopsies and injections, all of which contribute to clinically diagnosing and/or treating a patient [2]. These imaging modalities and procedures have become essential tools requested by GPs [3]. In Australia, within the Medicare Benefits Scheme, most tests and procedures are available to patients at little to no cost to them. As such, a surge in the number of diagnostic imaging conducted in 2022–23 has been shown (www.aihw.gov.au/reports/primary‐health‐care/medicare‐subsidised‐care‐2022‐23/contents/diagnostic‐imaging‐services). For example, in a quarterly Medicare data update provided by the Australian Government, diagnostic imaging services in Australia demonstrated a remarkable year‐on‐year increase of 11.3% during January–March 2023, compared to the same period in the previous year [3]. One possible explanation for this is the widespread availability of online health information. This encompasses public health campaigns [4], various media outlets (such as television and newspapers) [5], and smartphone software applications [6]. Such accessibility of health information allows patients to make more informed decisions about imaging studies and to make choices for themselves [7]. The rise of patient‐initiated imaging has raised concerns within the medical community around its appropriate use [8, 9, 10].

In Australia, only healthcare professionals like GPs, acting as gatekeepers, can provide a letter of request to radiology departments for scans or procedures to be performed on the patient. As gatekeepers, the GP's role includes the authority to decide on the most appropriate referrals to specialists, implement diagnostic workup, and coordinate extended care [11]. Patients often desire medical imaging tests based on information obtained through health advertising and/or accessed through their own online searches, requesting studies from their GP that they believe are needed. Patients requests for imaging can potentially devalue the gatekeeper role of GPs as primary caregivers [12]. On the basis of clinical assessment and expert knowledge, GPs may consider patients’ requests unnecessary. However, fulfillment of these requests may be seen as supportive of patient autonomy [13], resigning to patient pressure [14], and/or mitigating litigation fears [15].

Patients’ proactive management of their health through requests for referrals can be viewed as pursuing preventative medicine. However, when GPs consider these requests are not clinically indicated, they may encounter patient dissatisfaction when responding negatively [16]. This may even escalate to aggressive confrontation [14], particularly when the patient's desire for imaging is due to feelings of anxiety or if they consider it a form of a disservice [17]. Anecdotally, for example, patients who might fear a miscarriage during their pregnancy could ask their GPs for an ultrasound examination solely to alleviate their anxieties. Once Visualising the live heartbeat of the baby, they feel reassured. Another common scenario in the research literature concerns headaches, for which patients request computed tomography (CT) to rule out underlying pathologies such as brain cancer [18]. Guiding patients through evidence‐based medicine and using established imaging guidelines can allow GPs to manage patient care without needing to acquiesce to patients’ requests. However, this is not often enacted due to the pressure to fulfill requests from the patients or their family members [19].

Radiologists, on the other hand, have not traditionally been responsible for the direct management of patients. Instead, they act as a means to a patient's progression through the healthcare system [20]. Thus, much like laboratory services, radiology has been utilised as a readily accessible, relatively quick, minimally invasive modality for clinical diagnosis and treatment options [21]. Previous studies have identified challenges to the appropriate use of imaging within the healthcare team and with patients [22], barriers and facilitators to guideline adherence and imaging use [23], and the rising costs of imaging in Australia [24]. However, there is a scarcity of studies within Australia on the impact of imaging use on radiologists’ experiences and workload in particular, due to GPs’ fulfillment of patient requests.

Patient‐initiated requests have consequences that extend beyond the patient–GP relationship and involve a broader healthcare team. These include radiologists and other allied healthcare workers conducting or reporting on these examinations. The aim of our study was to assess the perceived impact of patient‐initiated medical imaging requests adding to radiologists’ workload. Our research aimed to explore (1) radiologists’ perception and experience with patient‐initiated imaging requests, (2) their experience with patients’ awareness of the merits and limitations of requested radiological studies, (3) the impact of unnecessary requests on radiologists’ workload, and (4) what strategies radiologists consider could be employed by GPs to mitigate unnecessary requests.

## 2 Methods

### 2.1 Design

A cross‐sectional survey study was conducted between November 2023 and February 2024 with radiologists working in the same company within private multi‐specialty medical facilities across Australia. Education coordinators of this company were recruited to distribute the survey via the company's intranet service. An educational coordinator was chosen to keep recruitment at arm's length from the researchers. There were approximately 100 radiologists working full‐time across the various sites when the recruitment flyer was sent. Upon clicking the embedded link, a participant information sheet explained the purpose of the study and asked if they were willing to volunteer. Once they accessed the online survey, radiologists could consent to participating and answer the survey questions. Radiologists could withdraw at any time during the study by clicking out of their browser.

### 2.2 Survey Instrument

The survey was comprised four sections, with a total of 10 questions:

- Section one explored whether radiologists had encountered patient imaging requests and any reasons they were aware of as to why patients requested imaging.
- Section two investigated the impact of patient imaging requests on radiologists’ workload and whether they felt pressured to finish reports quickly.
- Section three focused on their views regarding patient‐initiated requests, their experiences addressing patient‐anticipated outcomes, and the types of diagnostic investigations requested by patients.
- The fourth and final section asked radiologists about their perceptions of GPs’ compliance with patient demands. This section used fixed choices, including a sliding scale and a matrix table, and some open‐ended questions to enrich the data with more detailed responses.

The research team designed the survey based on similar studies in the literature [25, 26]. The draft survey was piloted by two radiologists to assess its readability and comprehensibility, and alterations were made to some questions based on their feedback. All responses were anonymous.

### 2.3 Participants

A total of 37 survey responses were received, indicating a response rate of 37%. Of those, 10 were incomplete, and 27 were analysed. The number of years participants had practiced as radiologists varied, with nearly half having over 20 years of experience (*n* = 14, 48%) and a quarter having less than 5 years of experience (*n* = 6, 21%).

### 2.4 Statistical Analysis

Descriptive statistics were used to assess radiologists’ perceptions of why patients made imaging requests and, from patients’ requests, the types of imaging most requested that were considered unnecessary by the radiologist. To understand the impact of clinically non‐indicated studies on radiologists’ workload, the cross‐tabulation of variables was used to identify patterns and relationships. Using responses from 27 radiologists through cross‐tabulation, the data were categorised by two key variables: (1) “The frequency of feeling pressured by GPs and/or patients to complete imaging reports quickly” and (2) “The impact of clinically irrelevant requests on their workload.” Results were analysed using IBM SPSS version 27.0 for Windows (SPSS Inc., Chicago, IL, USA).

### 2.5 Content Analysis

The open‐ended questions asked participants what imaging studies radiologists considered, in their opinion, were the most commonly requested by patients; whether patients were generally aware of the limitations and merits of procedures requested; what expectations patients had during interventional procedures where radiologists interacted directly with patients, such as musculoskeletal injections; and what strategies they believed GPs could employ to address patients’ requests.

There were 26 open‐ended responses from 37 radiologist respondents who provided a mean of 15 words (minimum of 5 and maximum of 55). Open‐ended questions were categorised using conventional content analysis [27]. Considering the limited research on this subject, preconceived categories were not possible. Instead, responses were categorised as they emerged from the data, and these categories were further developed into themes [27]. To do this efficiently, the data were uploaded to a Microsoft Excel worksheet (Microsoft Corporation, 2018). Data were open coded through an iterative process of reading and creating codes from extracted statements. Once codes were developed, the extracted statements were categorised under these codes. These categories eventually became themes that were mapped according to the research questions [28].

## 3 Results

### 3.1 Results From Multiple‐Response Questions

#### 3.1.1 Radiologists’ Experience With Patient‐Initiated Imaging Requests and Types of Requested Studies

**CHART 1:**
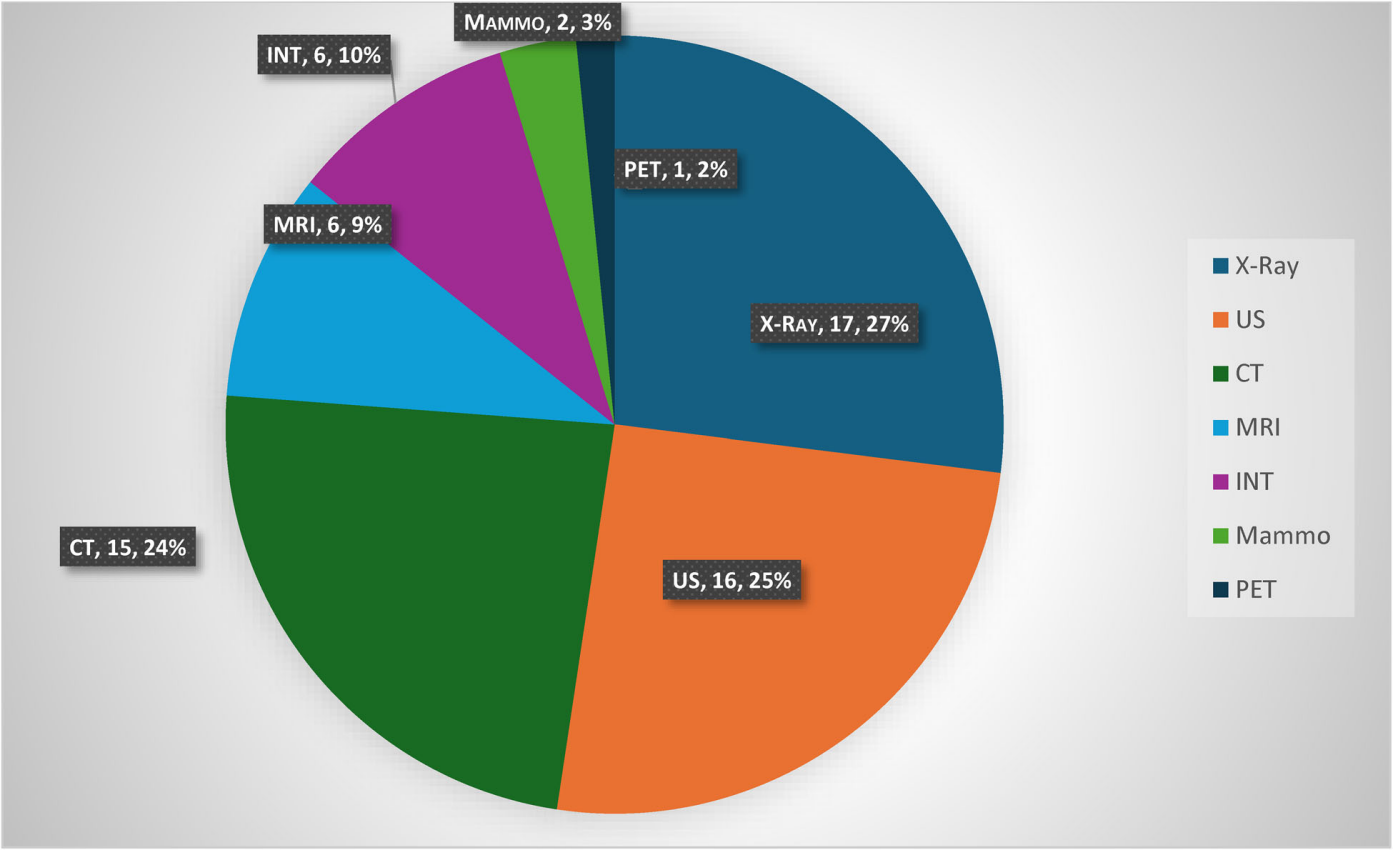
Non‐indicated imaging request types as reported by participants. INT, intervention; CT, computer tomography; MRI, magnetic resonance imaging; PET, positron emission tomography; Mammo, mammography; US, ultrasound.

A total of 18 (55%) participants agreed that they experienced patient‐initiated radiological imaging, as reported by GPs via telephone conversations or as noted on referrals. Radiologists reported X‐rays as being the most frequently requested non‐indicated studies (*n* = 17, 27%), followed by ultrasounds (*n* = 16, 25%) and CT scans (*n* = 15, 24%), with a scattering of other responses as indicated in Chart 1.

**GRAPH 1:**
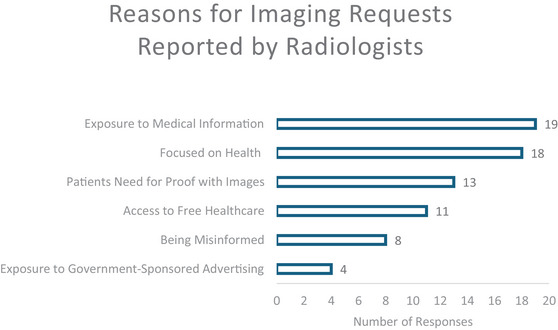
Reasons for imaging requests as reported by radiologists.

Participants reported they believed patients were requesting radiological scans and interventions due to exposure to medical information online and on social media or advice from friends and family (*n* = 19, 65.5%). Many indicated they thought patients were increasingly focused on health and wellness (*n* = 18, 62%) and sought imaging to provide proof of wellness (*n* = 13, 45%). To a lesser extent, radiologists stated that patients were more likely to want radiological studies because Medicare covered the cost, and therefore they felt entitled to these services (*n* = 11, 38%). They further noted they felt some patients were misinformed (*n* = 8, 28%) and/or influenced by government‐sponsored advertising (*n* = 4, 14%). This is shown in Graph 1.

When radiologists were asked to comment on what expectations patients bring into the consultation when having interventional procedures, most (*n* = 17, 63%) reported high expectations for immediate pain relief. Some (*n* = 5, 19%) reported that unrealistic expectations (including misconceptions about what the procedure can accomplish) led to disappointment.

#### 3.1.2 The Impact of Unnecessary Requests on Radiologists’ Workload

Anecdotally, in an average radiologist's week of reporting approximately 700 radiological studies, six participants considered about 24% of imaging to be unnecessary. In a cross‐tabulation analysis, it was observed that nearly a third of radiologists (*n* = 8, 30%) experienced pressure “most of the time” to expedite report completion to align with patient demands. The pressure was also from GPs’, who wanted to deliver prompt services to their patients. At the same time, the remaining respondents (*n* = 19, 70%) did not acknowledge this pressure.

When respondents were asked “why do you think GPs fulfil patient requests?,” in a multiple‐response question (more than one response available), the majority of radiologists surveyed believed this was because the patients were health‐anxious (*n* = 20, 69%), GPs were unable to argue with patients (*n* = 17, 58.6%), or they feared litigation in the event of a missed diagnosis (*n* = 16, 55.2%). Most (*n* = 19, 70%) participants reported that they did not believe patients were aware of the merits and limitations of the requested imaging tests.

### 3.2 Results From Open‐Ended Questions: Unrealistic Expectations

Radiologists proposed several strategies to avert unnecessary scanning for GPs and radiologists. These included GPs discussing the risks and benefits of tests with patients and radiologists doing the same in cases where they were consultating with patients for interventional studies. Further, they suggested healthcare providers must be made aware of the necessity of diagnostic tests and clarify the limitations of clinically non‐indicated requests and that when patients demand imaging studies, request fulfillment by GPs can result in ambiguous ordering. Participants stressed the importance of a clear statement of the clinical indications for a study on the imaging referral so that they can correlate the tests requested with the GP's clinical concerns. Radiologists suggested GPs should place greater emphasis on reviewing patients’ clinical presentation using the “watch and wait” strategy discussed in the literature to give the symptoms time to pass without any interactions [29]. This strategy can be combined with a safety plan if the symptoms do not resolve [29, 30]. Radiologists commented that building rapport with the patient and ensuring continuity of care are essential to patient‐centered care, and that unrealistic expectations could be addressed with appropriate discussions with patients about the outcomes of procedures. They also suggested developing guidelines may be useful for GPs, outlining accepted standards of practice when patients request imaging. Radiologists suggested that a reimbursement structure be established with Medicare so that when they consult patients regarding imaging requests from their GPs, they are compensated for the time spent on these discussions. The full list of suggestions is provided in Table 1.

**TABLE 1:** Radiologists’ proposed strategies to mitigate unnecessary requests.

Strategy	Suggestions
**Patient education**	Emphasise the importance of health providers explaining procedures to their patients, discussing risks and benefits, and educating them on the recommended diagnostic tests’ indications and limitations
**Follow‐up care**	Health providers review their patients’ condition (such as presenting signs and symptoms) before requesting imaging and/or following up with patients to see if symptoms resolve
**Building rapport**	Health providers should establish continuity of care and build rapport to provide patient‐centered care, enabling productive discussions around imaging requests
**Establishing guidelines**	Develop guidelines by the organisation and/or professional bodies for all health providers to address patient‐requested procedures to avoid unnecessary imaging
**Medicare rebates**	Radiologists propose that Medicare provide rebates for consultations to reimburse the time spent on discussions, enabling informed conversations among radiologists, GPs, and patients

## 4 Discussion

Past studies reflect that GPs face several challenges when addressing patient‐initiated imaging [23]. Requests from patients may result in GP's nonadherence to existing guidelines through the desire to fulfill requests, for several reasons [Walderhaug et al. (2022) suggested various approaches for GPs to address patients’ requests, highlighting the “watch and wait” strategy, where GPs ask the patients to give the symptoms time to pass without intervention, and often combining this strategy with a follow‐up plan should symptoms remain unresolved [30]. They further suggested that GPs ease patient anxiousness through reassuring, normalising, recognising symptoms, giving patients confidence in their clinical assessment, and finding effective ways to say “no” [30]. Gransjøen et al.’s study (2018) addressed GPs and radiologists’ perspectives on patient imaging requests. They recommended easy‐to‐access and comprehensible guidelines be made available to GPs to advise their patients on the merits and limitations of the requested study. Their study also indicated that radiologists preferred more informal knowledge exchanges with GPs than adopting formal guidelines on the merits and limitations of tests and procedures [23]. However, considering that each discipline often works within its silos of practice, discussions around guidelines across the various disciplines can be limited due to a lack of communication.

Our study explored radiologists’ perspectives on patient requests for diagnostic imaging and GPs fulfilling such requests. We sought to understand the experiences and perceived consequences of patient‐initiated imaging on radiologists and propose strategies for mitigating unnecessary scanning to ease the burden on their workload. Our private clinic radiologist participants identified several factors they perceived as driving patient requests, including exposure to online medical information, the need for proof of wellness, and their belief that most requests came at little to no cost under the Australian Medicare Scheme. Radiologists also expressed that patients were largely unaware of the merits and limitations of imaging studies and agreed that the requested imaging studies were often unnecessary. However, radiologists reported an increased workload linked to satisfying both GPs and patients by completing reports quickly. They also identified that patients had many expectations, particularly during interventional studies where there is direct radiologist‐patient contact, and expectations were high for immediate therapeutic benefits, particularly with musculoskeletal injections.

Our study showed over half of the surveyed radiologists have encountered patient‐initiated imaging requests, this being advised by a GP on a request or by a phone call. The more commonly requested imaging modalities included X‐ray, ultrasound, and CT. Maskell (2022) found that the most critical overall reason for the increased usage of radiological investigations was the need for diagnostic accuracy. Moreover, imaging was a better option for GPs as it was feasible, rapid, inexpensive, and easily accessible [31]. They added that radiologists in their study agreed that managing radiology use by health professionals such as GPs is challenging, as it often depends on the GPs’ knowledge, beliefs, training, and practicing methods [31]. This trend is reflected in our study, where the predominant choices are easily accessible, readily available, and primarily covered under the Medicare rebate schedule [3].

In the short response section, radiologists expressed that patients’ expectations were high when they underwent interventional procedures, particularly musculoskeletal injections. Radiologists in our study expressed that patients were hoping for immediate pain relief. Little et al. (2004) found that the most significant independent predictor of GPs’ behavior was their belief that patients arrived with specific expectations, leading them to feel obligated to meet those needs. This included prescribing medications, conducting examinations, running tests, and referring patients to specialists. However, the study also revealed that patients did not always seek referrals or prescriptions; rather, they primarily wanted their concerns to be acknowledged and addressed. A majority of GPs in their study believed that prescribing medical services because of pressure from the patient was unnecessary. Their study concluded that GPs perceived beliefs about patients’ expectations or pressure were far more significant than patients’ actual expectations from GPs, encouraging GPs to question patients' expectations at the start of the consultation [33].

In responding to the survey question “Why do you think GPs request scans that are clinically non‐indicated?,” the majority of participants perceived that GPs felt it was necessary to appease patient anxiety—agreeing that by consenting to patients’ requests, they also would deter any litigation concerns, particularly in the event of missing an underlying pathology. Though appeasing patients with imaging can be seen as a contraindication, several complexities arise when addressing a patient holistically. These include the urgency of the situation, the limited time during consultation, and the patient's past medical history. However, Walderhaug et al. (2022) found that GPs in their study were confident in declining requests for imaging, stating that they explained their diagnostic approach to patients, often thinking aloud their reasoning before coming to a negative response. They reasoned that such an approach was favourable as it reflected empathy towards the patient through open communications and transparency and facilitated GPs to ask for a detailed clinical history. Ottenheijm et al. (2014) further emphasised that patients were likely to place greater confidence and trust in GPs who were transparent in their diagnostic approach. Such an approach could likely be a foundation for a long‐term therapeutic relationship where patients would be more receptive to GP recommendations [19].

We also found that radiologists thought that patients’ exposure to online medical information, media portrayal of healthcare, and advice from friends and family play a crucial role in patients’ decisions to request imaging studies. Wong & Cheung (2019) found that the majority of their participants used the internet to seek online health information and chose websites based on convenience rather than accuracy or authoritativeness [34]. However, when poorly informed patients request imaging with no clinical merit, denying them the request can pose several challenges [15]. In addressing these issues, Nilsen & Malterud (2017) found that GPs experienced several challenges in declining patients’ requests. Many were confronted by patients who openly disagreed with them in anger or sadness and, in some cases, displayed open aggression [35]. Radiologists in our study indicated that patients came with high expectations for immediate pain relief from interventional procedures, sometimes having unrealistic expectations. If patient expectations were not met or if the procedure requested was not carried out, such outcomes were met with disappointment, leading to various negative emotional responses from patients. These findings underscore the complexity of managing patient agency, highlighting the need for patient education and communication strategies to manage expectations [4].

Because radiologists are part of the specialised care arm in the health context and act primarily as consultants rather than direct patient managers, their role in interpreting studies is critical for GPs. The rising demand for imaging, driven by patient expectations, suggests a need for radiologists to collaborate more closely with GPs to ensure appropriate use of resources. When responding to the question, “What do you think are strategies GPs could use to reduce inappropriate patient requests?”, our respondents recommended that GPs adopt patient education around imaging. This could be done by implementing guidelines when a patient requests non‐indicated imaging. Integrating evidence‐based medicine with clinical and patient preferences has proven valuable when avoiding unnecessary scanning [16]. This is particularly so when imaging requests are considered non‐medically indicated, as expressed by radiologists in our study, as there is a potential for increased healthcare costs and harmful downstream interventions in the event of false‐positive incidental findings [22]. These incidental findings would not have caused harm if left undiscovered. Moreover, in the case of false‐positive results, it raises unnecessary alarm and anxiety for patients, leading to more testing (wiserhealthcare.org.au).

## 5 Limitations

Although the response rate is modest, it reflects typical survey participation rates in specialised healthcare settings across Australia [36]. In our studies, these responses were from radiologists residing in Queensland, New South Wales, Victoria, South and Western Australia. Many of these radiologists found it difficult to volunteer for this research study because they often had very busy schedules; as such, the results from a small sample size cannot reflect the views of radiologists across complex medical practices within Australia. This also limited their engagement in intranet activities, which limited responses to our survey as it was only available on the intranet. Moreover, some radiologists might hold specific views on patient‐centered requests, perceiving them as trivial. This perception could result in their decision to avoid participating in the survey. The diverse levels of experience among respondents, with nearly half having over 20 years of practice, lend credibility to the findings, with the study being the first to examine radiologists’ views of patient‐initiated diagnostic imaging. The sample consisted solely of radiologists in private practices within a specific company in Australia. Further studies could be undertaken to understand specialists and subspecialists in both medical and surgical fields. Future research with a larger sample size across public, private, and international settings could be explored to enhance generalisand ability and consider dichotomous variable of patient and non‐patient generated requests affecting radiologists’ workload. This study focused on the views of radiologists and did not capture the views of GPs and patients.

## 6 Conclusions

This study addressed our four key aims regarding radiologists’ perspectives on patient‐initiated imaging requests, which has significant consequences for radiologists and the broader healthcare system. To understand our first aim, which is to explore radiologists’ perceptions and experiences with patient‐initiated imaging requests, our survey showed that respondents mainly encountered patient requests through GPs indicating patient requests study “X” in their referral letters or during phone conversations. They also expressed significant concerns about the increasing trend of patient‐initiated requests. Most participants perceived that these requests were driven by the availability of online health information and patients’ desire for proof of wellness. In addressing our second aim, radiologists confirmed many patients lacked knowledge of the merits and limitations of the studies they requested. However, these respondents perceived pressure to promptly accommodate GPs and patients’ imaging needs. Thus, we can provide evidence confirming the third aim of this study, whether unnecessary requests impacted radiologists’ workload, showing that the impact of unnecessary imaging extends beyond the patient–GP relationship to radiology services. This is specifically where radiologists experience pressure to complete their work quickly and are burdened by an increased workload. Most radiologists considered GP's compliance was due to patients’ health anxiety, GPs' inability to argue with their patients, and litigation fears. Lastly, we asked our respondents what strategies radiologists might consider could be employed by GPs to mitigate unnecessary requests, and our participants suggested clear communication, thorough medical examination, shared decision‐making, and building trust to prevent unnecessary imaging. This includes ongoing collaboration with radiology team members so GPs can be updated on the types of tests and treatments that would most benefit patients. These findings, we believe, underscore the tension between patient autonomy and clinical decision‐making. To address this, healthcare policymakers might consider developing more explicit interdisciplinary guidelines that facilitate better collaboration between GPs and radiologists in managing patient‐initiated imaging requests. These guidelines could reduce unnecessary imaging and promote evidence‐based practice.

## 7 Practice Implications

The study underscores the importance of clear, evidence‐based guidelines to support GPs in responding to patient pressures for unnecessary imaging tests. An important aspect of implementing guidelines is educating patients and guiding them through the merits and limitations of imaging studies and procedures. This could be achieved through improved communication strategies among radiologists, GPs, and patients to foster a collaborative approach to imaging requests, which can help ease the workload on radiologists.

## Author Contributions

**Lizzie De Silva:** Conceptualisation, Data Curation, Visualisation, Writing Original Draft Preparation. All other authors: Resources, Supervision, Validation, Formal analysis, Review & Editing.

## Ethics Statement

The University of Sydney Human Research Ethics Committee (HREC) Project number 2023/831. Approval Period: 15/08/2022–15/08/2026.

## Conflicts of Interest

The authors declare no conflicts of interest.

## Data Availability

Data are available upon request.
